# Biofilm formation by *Bacillus subtilis* is altered in the presence of pesticides

**DOI:** 10.1099/acmi.0.000175

**Published:** 2020-11-12

**Authors:** Rachael Newton, Jennifer Amstutz, Joyce E. Patrick

**Affiliations:** ^1^​ Truman State University, 100 E Normal Kirksville, MO 63501, USA

**Keywords:** plant growth-promoting bacteria, *Bacillus subtilis*, swarming, biofilm formation, pesticide, neem

## Abstract

*
Bacillus subtilis* uses swarming motility and biofilm formation to colonize plant roots and form a symbiotic relationship with the plant. Swarming motility and biofilm formation are group behaviours made possible through the use of chemical messengers. We investigated whether chemicals applied to plants would interfere with the swarming motility and biofilm-forming capabilities of *B. subtilis in vitro*. We hypothesized that pesticides could act as chemical signals that influence bacterial behaviour; this research investigates whether swarming motility and biofilm formation of *B. subtilis* is affected by the application of the commercial pesticides with the active ingredients of neem oil, pyrethrin, or malathion. The results indicate that all three pesticides inhibit biofilm formation. Swarming motility is not affected by the application of pyrethrin or malathion, but swarm expansion and pattern is altered in the presence of neem oil. Future studies to investigate the mechanism by which pesticides alter biofilm formation are warranted.

## Data Summary

The authors confirm that all supporting data, code and protocols have been provided within the article or through supplementary data files.

**Impact Statement**

This research investigates a previously unexplored aspect of bacteria–plant symbiosis. Many pesticides are available to the home gardener, with a variety of active ingredients. Our research aims to discover if these pesticides, applied in the hope of promoting plant growth, could be interfering with microbial behaviours necessary to establish beneficial plant–microbe interactions. Many studies have investigated changes in bacterial metabolism in soils in response to pesticides, but none have looked at changes to specific multicellular behaviours. This research should be of interest to those interested in environmental microbiology, plant–microbe interactions and bacterial behaviours. This work represents an incremental advancement in our understanding of how agricultural chemicals influence bacterial behaviour.

## Introduction

Plant growth-promoting bacteria (PGPB) are bacteria that form specific symbiotic relationships with plants and enhance plant growth through a variety of means [1–4]. Many of the PGPB exist in the rhizosphere, the soil within a few millimetres of the plant root surface [5]. PGPB perform various activities to aid plant growth, including nitrogen fixation, siderophore production to reduce iron toxicity to plants and inhibit pathogen growth, production of indolic compounds, reduction of ethylene gas production by stressed plants through ACC deaminase and phosphate utilization [2]. *Bacillus* spp. are PGPB that have been reported to colonize plant roots and exist in a symbiotic relationship with the plant, promoting growth in maize via siderophore production and nitrogen fixation, and rice via ACC deaminase, among other mechanisms [2–9]. Growth promotion by *Bacillus* species requires colonization of plants, either externally on roots or as endophytes [2, 6]. Colonization of plant roots with *Bacillus subtilis* requires both swarming motility and biofilm formation, as well as chemotaxis [6–8].

Swarming is a coordinated bacterial motility that allows rapid migration over and colonization of a surface [10]. Swarming by the undomesticated *B. subtilis* strain NCIB 3610 requires high cell density and the production of the wetting agent surfactin [11, 12]. Surfactin production is activated by the quorum-controlled response regulator ComA and cells mutated for ComA are nonswarming, but swarming can be restored with the addition of surfactin [13–16] (Patrick and Kearns unpublished data). Thus, extracellular signalling is important for swarming behaviour. Indeed, it has been shown that there are chemical signals between plant roots and the colonizing *B. subtilis* [6, 17, 18].

*B. subtilis* also has the ability to form a biofilm, which results in the aggregation of bacteria on a surface and the production of an extracellular matrix to hold cells in place and form a physical barrier [18–20]. In the laboratory setting, when *B. subtilis* was introduced to roots of *Arabidopsis thaliana*, it formed a biofilm on the root surface [8, 21, 22]. *B. subtilis* colonization of plant roots results in induced systemic resistance (ISR) in the plant, and has been demonstrated in several plant species [23, 24]. Surfactin may be partly responsible for the stimulation of ISR by *B. subtilis* [24]. In addition, the formation of a biofilm around the root could provide a physical barrier against bacterial pathogens that might be harmful to the plant [25].

Multiple studies have investigated the effects of pesticides on soil microbial composition [26–28]. Many focus on bacterial abundance and diversity in soil, while a few measure changes in metabolic activity [29–32]. However, we were unable to locate studies that directly assessed the impact of pesticides on bacterial behaviours, such as swarming motility and biofilm formation. Given that these behaviours are essential for the protective activity garnered by *B. subtilis* and other PGPB, studying the impact of pesticides on these behaviours seems relevant.

We wondered whether the application of naturally derived pesticides – such as neem oil or pyrethrins – or synthetic pesticides – such as malathion – applied in the hopes of promoting plant growth through insect control, could actually be disrupting the chemical signalling required for plant root colonization, and therefore indirectly hindering the growth of the plant by preventing biofilm formation or swarming motility by protective *B. subtilis *. Specifically, we asked if these pesticides could interfere with the swarming motility or biofilm-forming behaviours of *B. subtilis in vitro*.

## Methods

### Strains, media and growth conditions

*B. subtilis* NCIB 3610 (a generous gift from Daniel Kearns) was used for all experiments. Unless otherwise specified, *B. subtilis* cultures were grown from frozen stocks on Luria–Burtani (LB) plates (10 g Bacto tryptone, 5 g Bacto yeast extract, 5 g NaCl l^−1^) solidified with 1.5 % Bacto agar at 37 °C. Liquid cultures were produced by subculturing a single colony from the LB plates into LB broth, and were agitated in a New Brunswick Scientific Excella E24 shaking incubator at 150–200 r.p.m. at either 27 or 37 °C as specified in each experiment. For floating pellicle and colony architecture biofilm assays, *B. subtilis* strain 3610 was grown in minimal salts glutamate glycerol (MSgg) broth (95.37 ml sterile MQ water, 7.5 ml 0.1 M phosphate buffer, 30 ml 0.5 M MOPS pH 7, 3 ml 0.1 M MgCl_2_, 1.05 ml 0.1 M CaCl_2_, 0.75 ml 0.01 M MnCl_2_, 0.9 ml 8.35 mM FeCl_3_, 0.15 ml 1.0 mM ZnCl_2_, 0.03 ml 0.01 M thiamine HCl, 1.5 ml 50 % glycerol, 7.5 ml 10 % glutamic acid, 0.75 ml 10 mg ml^−1^ tryptophan, 0.75 ml 10 mg ml^−1^ phenylalanine and 0.75 ml 10 mg ml^−1^ threonine 150 ml^−1^ media) or on MSgg plates supplemented with 1.5 % Bacto agar. All media components were made as solutions in MQ water and sterilized by either autoclaving or filter sterilization, and aseptically mixed immediately before use. For the swarming assay, *B. subtilis* was inoculated onto plates containing 25 ml of LB broth solidified with 0.7 % Bacto agar that had been prepared the day before, allowed to solidify at room temperature overnight and had been let to dry for 20 min open-faced in a laminar flow hood. Pesticides were added into or as a drop onto the agar as appropriate. A phosphate-buffered saline (PBS)/India ink buffer (5 µl India ink, 1 ml 1× PBS buffer) was used for cell resuspension. When appropriate, pesticides were included in the plates in the following concentrations: 10 µl working dilution, 10 µl 1 : 10 dilution and 10 µl 1 : 100 dilution 25 ml^−1^ media.

### Pesticide dilution and usage

The pesticides – neem oil (Southern Ag Triple Action Neem Oil), pyrethrin (Miracle-Gro Nature’s Care 3-in-1 Insect, Disease and Mite Control) and malathion (Spectracide Malathion Insect Spray) – were purchased from a local home and garden supply store. When the pesticide was a concentrate, the concentrated pesticides were diluted in sterile water according to the manufacturer’s directions to produce the ‘working dilution’. From these working dilutions, samples were further diluted to 0.1× and 0.01× in sterile ultrapure water. For growth curve and biofilm assays, working, 0.1× and 0.01× dilutions of pesticide were added to liquid media at a concentration of 1 : 1000 (25 µl ml^−1^ broth). For swarm assays, a 10 µl drop of pesticide was applied to the surface of the media, or was added to molten agar (10 µl 25 ml^−1^) prior to pouring plates. Alternatively, 100 µl was spread over the surface of the agar with sterile glass beads, prior to the 20 min drying time.

### Floating pellicle biofilm assays

Cultures of *B. subtilis* were inoculated into 2 ml of LB broth and grown overnight in a shaking incubator at 27 °C and 150–200 r.p.m. MSgg media was prepared from sterile stock solutions on the day of use and pesticides were added as appropriate. Ten millilitres of MSgg broth were aliquoted into each well of six-well culture plates. Ten microlitres of overnight culture were pipetted into each well. The plates were incubated for 1 week in a dark, undisturbed area at 25 °C. Wells were photographed with the lids removed at 24, 48, 72 and 168 h against a black background. Observations of the location and architecture of the resulting pellicle were recorded.

### Crystal violet biofilm assays

Measurements of biofilm density were made using a modification of the method of O’Toole and Kolter [33]. Ten microlitres of a turbid overnight culture was added to 10 ml of MSgg media and mixed via vortex. One hundred microlitres of this cell suspension was pipetted into the wells of a 96-well microtiter plate. Wells with MSgg only served as controls. Two and a half microlitres µl of each dilution of pesticide (see above for dilutions) was added to the wells. The plate was incubated at 25 °C for 96 h. After incubation, the culture was dumped out and the plate was rinsed three times by submersion in a tub of distilled water. After each rinse, the water was dumped out, and the plate was inverted and tapped dry on paper towels. One hundred and fifty microlitres of 0.1 % crystal violet solution was added to each well and allowed to incubate at room temperature for 15 min. The crystal violet was dumped out and the plate was rinsed and dried as before. The plate was allowed to sit inverted for ~1 h to dry. Two hundred microlitres of 95 % ethanol was pipetted into each well to solubilize the crystal violet. The plate was read at 540 nm on a Thermo Scientific Multiskan MCC plate reader using Ascent software. The absorbance values for five wells for each condition were averaged and compared to controls using a two-tailed *t*-test. Significant differences between dilutions of pesticide were tested using analysis of variance (ANOVA). Significance was determined at the 95 % confidence interval.

### Colony architecture biofilm assays

MSgg agar plates were prepared the same day of use with pesticides added as described above. A sterile wooden inoculating stick was used to inoculate two to three colonies of *B. subtilis* as single dots on each plate. The plates were then incubated for 1 week in a dark, undisturbed area at 25 °C. Plates were photographed with the lids removed at 168 h using a Leica EZ4 W Stereomicroscope and Leica AirLab Image Capture Software.

### Growth curve assays

Cultures of *B. subtilis* were inoculated into 2 ml of LB broth and grown overnight in a shaking incubator at 37 °C and 150 r.p.m. The optical density of each culture was measured at 600 nm (OD_600_) using a Spectronic 200 Spectrophotometer. Twenty-five millilitres of sterile LB media in a 250 ml baffled flask were inoculated with enough overnight culture to produce a calculated OD_600_ of 0.01. Each flask was placed into the shaking incubator at 37 °C and 150 r.p.m. The OD_600_ of each culture was determined by removing 0.8–1 ml every 30 min for optical density measurements.

### Swarm expansion assays

Swarming motility assays were conducted using the Kearns method [11]. Cultures of *B. subtilis* were inoculated into 2 ml of LB broth and grown overnight in a shaking incubator at 27 °C and 150 r.p.m. Two hundred microlitres of overnight culture was subcultured into new tubes containing 2 ml of sterile LB media. In experiments where cells were pretreated, 0.8 µl of pesticide at the appropriate dilutions was added to the 2 ml of LB in the subculture tube prior to incubation. Cultures were grown at 37 °C at 150–200 r.p.m. for ~1–2 h. The optical density of each culture was measured at 600 nm (OD_600_). When the cultures reached mid-exponential phase (OD_600_ of ~0.5–1.0), 1 ml of cells was harvested by centrifugation and resuspended to a calculated OD_600_ of 10.0 in sterile 1× PBS plus 5 % India ink (Higgins). LB swarm agar plates (25 ml of LB broth solidified with 0.7 % Bacto agar that had been prepared the day before) were dried for 20 min open-faced in a laminar flow hood. Ten microlitres of resuspended cells was spotted onto the centre of an LB swarm agar plate and allowed to dry for 10 min open-faced in a laminar flow hood. Each plate was placed in a walk-in incubator agar side down at 37 °C. A straight line was drawn along a radius on the bottom of the plate and rate of swarming was measured against the line every 30 min until the cultures either reached the edge of the plate or stopped swarming for three consecutive measurements. After the assay, the plates were left in the incubator overnight and checked in the morning for any changes in swarm radius.

Two-factor generalized linear models were used to simultaneously test the effect of pesticide treatment and time, and the interaction of pesticide treatment and time on swarm expansion for each pesticide. Swarm radius was expected to change with time, and thus significant regression effects were ignored. Evaluation of the interaction effect for each model allowed us to determine if colonies exposed to different treatment levels within an experiment expanded at different rates over the time course of measurement; e.g. a significant interaction effect indicated that colonies generally expanded at different rates without pinpointing particular pairwise differences in expansion rate. Evaluation of the treatment effect for each model allowed us to determine if colonies exposed to different treatment levels within an experiment attained different average swarm size. Differences in average swarm size might be explained by different expansion rate or different timing of the initiation of the swarm. Statistical differences were determined using the alpha=0.05 significance level.

## Results

### Neem oil inhibits floating pellicle biofilm formation at high concentrations

The three pesticides used in this experiment were chosen because they are all readily available for the home gardener and commonly used. Malathion and pyrethrin represent two of the top four most commonly used insecticide active ingredients in the home and garden market sector in the USA [34]. All pesticides used were in their commercially available formulations, as would be used by the home gardener, so that their effects as well as their formulation ingredients could be observed [29]. Neem oil is approved for use in organic gardening [35]. Pyrethrins are derived from chrysanthemum flowers and are natural insecticides [36]. Malathion, however, is a synthetic organophosphate insecticide that is used both indoors and outdoors to control mosquitoes, ticks, fleas and other insects [37].

**Fig. 1.**
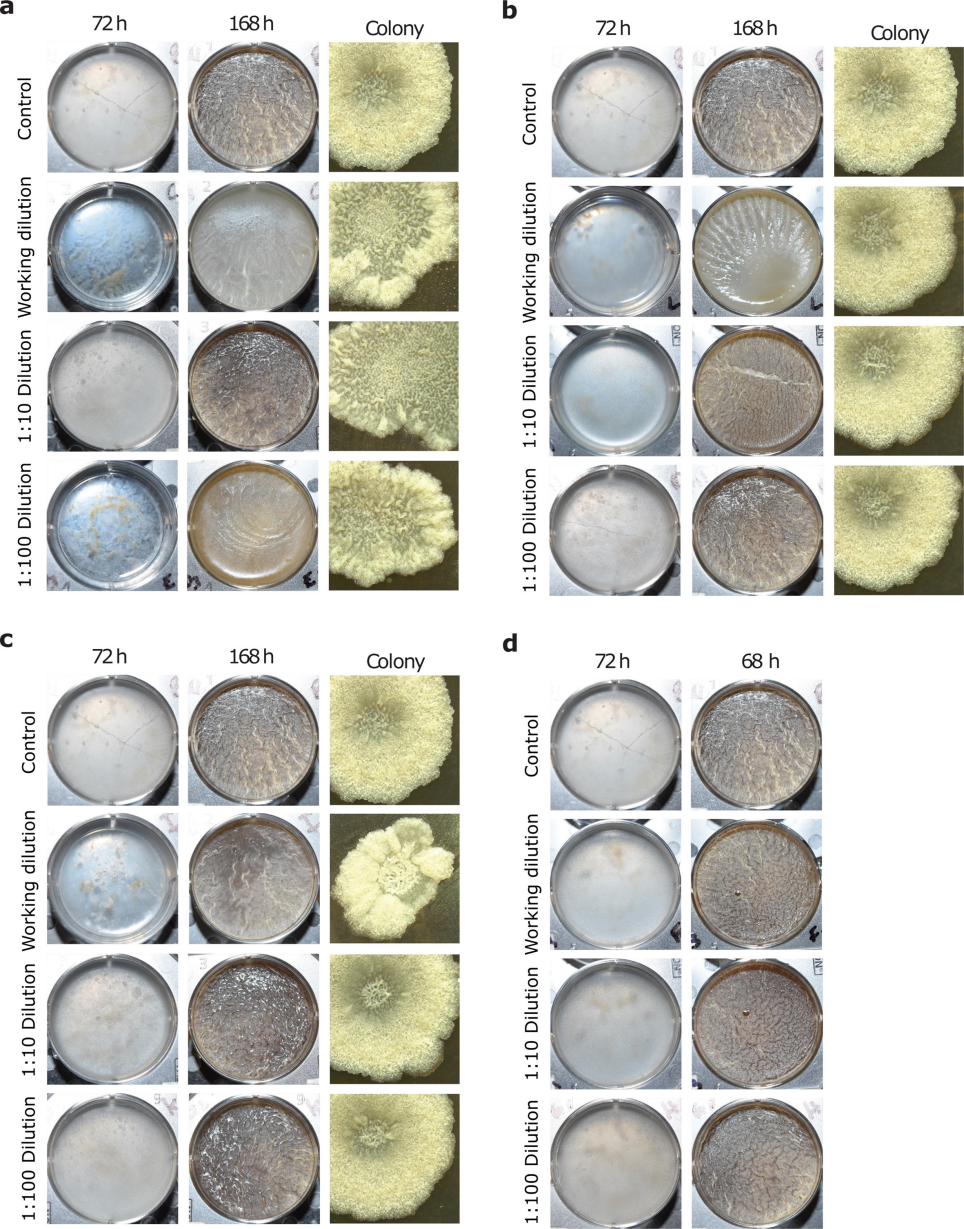
Neem oil alters colony architecture. Neem oil (a), pyrethrin (b), malathion (c) and mineral oil (d) at various dilutions were added to MSgg media. Biofilms were allowed to develop at 25 °C for 1 week and photographed from above against a black background.

We wanted to determine if any of the tested pesticides would alter biofilm formation, a protective behaviour important for colony survival and plant root colonization [23, 24]. We therefore conducted floating pellicle biofilm assays in liquid media using the robust biofilm-forming undomesticated *B. subtilis* strain NCIB 3610 in the presence of either neem oil, malathion, or pyrethrin. Pellicles appeared unaltered except in the presence of the highest concentration of neem oil tested (Fig. 1a). After 72 h, the pellicle had not formed, and cells appeared as clumps at the bottom of the plate (Fig. 1a). Compared to the control, the wells containing neem oil were slower to form a biofilm, and the resulting biofilms were less textured (Fig. 1a). Similarly, surface biofilms, assayed by colony architecture on agar plates, were altered. The central region of the colony that contains dense hills and valleys on control plates was observed to be expanded outward, closer to the edge of the colony when grown in the presence of any concentration of neem oil (Fig. 1a). Similarly, the texturing of the perimeter of the colony was greatly reduced in the presence of neem oil (Fig. 1a).

It is possible that neem oil inhibited pellicle formation by preventing cell aggregation. However, biofilm formation might have been inhibited due to a reduction in cell growth. In order to rule out the latter possibility, we conducted growth curves in the presence and absence of the pesticide. Neither growth rate nor yield was altered in the presence of neem oil (Fig. S1, available in the online version of this article). We conclude that neem oil inhibits biofilm formation at high concentrations by preventing cell aggregation at the liquid–air interface.

**Fig. 2.**
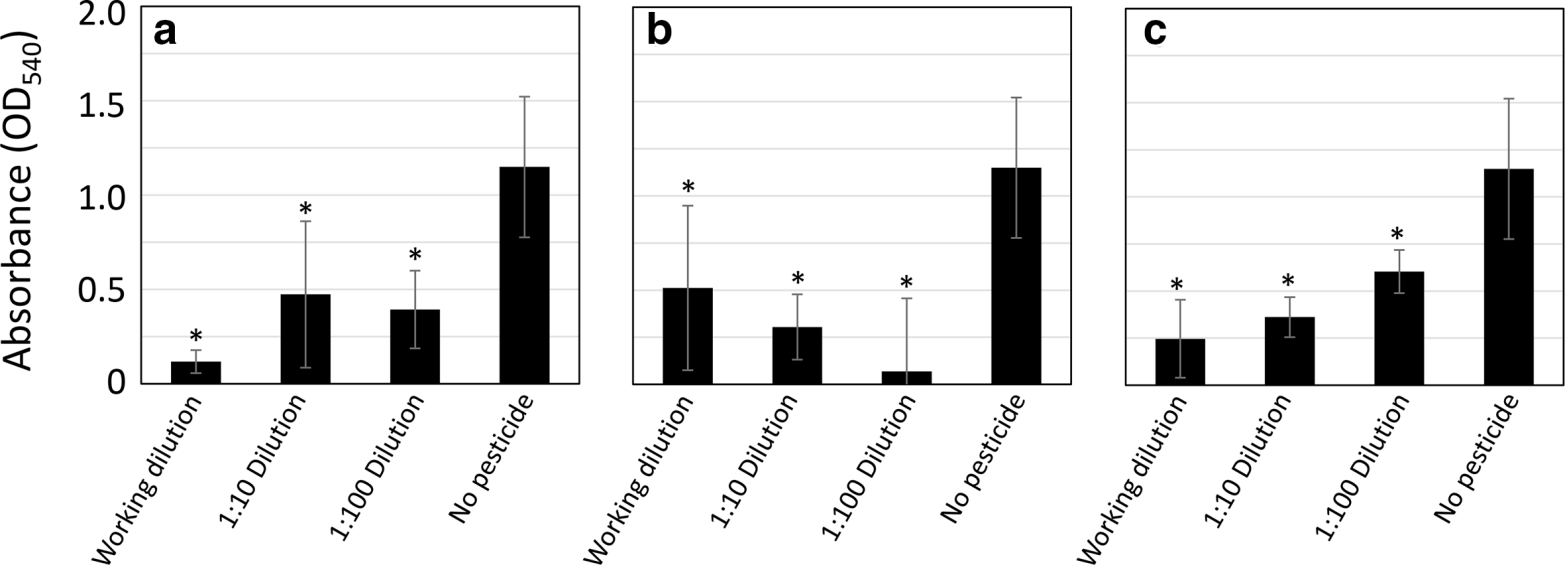
Pesticides inhibit biofilm formation in a microtitre crystal violet assay. Neem oil (a), pyrethrin (b) and malathion (c) at various dilutions were added to MSgg media in a 96-well microtitre plate. Biofilms were allowed to develop at 25 °C for 96 h and pellicle adherence to the sides of the plate was assayed using crystal violet staining and quantification using a microplate reader at 540 nm. All bars represent the average absorbance of five wells. Error bars represent the standard deviation of the mean. *, statistically significant difference from controls without the addition of pesticide, using a two tailed *t*-test, 95 % confidence interval. Within each group, no significant difference was found between dilutions of neem oil or pyrethrin using an ANOVA; 95 % confidence interval. Significant differences between the concentrations of malathion were found.

While the floating pellicle assay allows for a visualization of biofilm architecture, it does not provide a quantitative measure of biofilm formation. In order to quantify biofilm formation, we performed crystal violet biofilm assays. Compared to controls without pesticide, neem oil significantly inhibited biofilm formation at all concentrations tested (Fig. 2). However, the inhibition was not dose dependent, as there was no significant difference in the amount of crystal violet retained at any of the tested dilutions.

### Oil does not alter pellicle formation

We wanted to better understand how neem oil could be inhibiting floating pellicle formation. We hypothesized that the lipid nature of the neem oil might be preventing cell aggregation at the liquid–air interface necessary for pellicle formation. To further investigate this, we performed floating pellicle biofilm assays again, this time using mineral oil, which is commonly used as an overlay in microbiological experiments. Mineral oil was diluted to the same concentrations as neem oil and added to MSgg for a floating pellicle assay. The biofilms formed in this assay were indistinguishable from controls (Fig. 1d). We conclude that the lipid nature of the neem is not responsible for the delay in biofilm formation.

### Malathion and pyrethrin inhibit biofilm formation

We next tested the other two pesticides for their ability to inhibit biofilm formation. Pellicle formation in the presence of pyrethrin was slightly reduced at early time points compared to controls, but appeared to be similar to controls later in the assay. Only at the highest concentration of pyrethrin was there a slight change in the texture of the floating pellicle. However, in quantitative crystal violet assays, there was a significant reduction in biofilm formation in the presence of pyrethrin at 96 h (Fig. 2). As expected, the growth rate in the presence of pyrethrin was only slightly reduced early in the assay, but the yield remained the same, suggesting that the delay in pellicle formation was due to a delay in growth (Figs 1b and S1). Colony architecture in the presence of pyrethrin appeared to be unchanged (Fig. 1b).

Malathion exerted a minimal but noticeable effect on floating pellicle formation, but only at the highest concentration tested (Fig. 1c). In addition, in quantitative crystal violet assays, malathion reduced biofilm formation in a dose-dependent fashion (Fig. 2). The colony architecture on MSgg plates containing malathion was altered at high concentrations. At the highest concentration tested, malathion resulted in a smaller colony, with an irregular edge. The central hill and valley region was slightly enlarged. When the malathion was diluted, however, the effect on colony architecture went away (Fig. 1c). Malathion had no effect on growth rate or yield (Fig. S1). We conclude that all three pesticides have the potential to reduce floating pellicle biofilm formation.

### Neem oil acts as a chemorepellent and impairs swarming motility

**Fig. 3.**
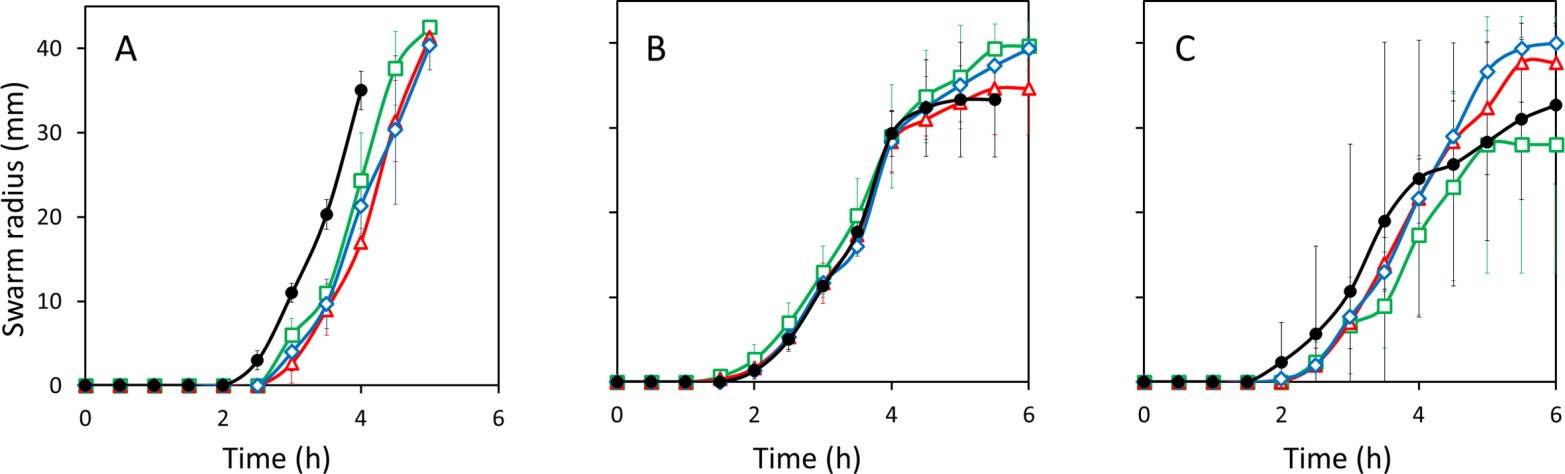
When applied as a drop to swarm plates, neem oil acted as a chemorepellent. Neem oil (a), pyrethrin (b) and malathion (c) were applied as a drop in various concentrations onto a swarming plate and the colony radii toward the drops were measured every 30 min. All lines represent the average of three replicates. Error bars represent twice the standard error of the mean. Neem oil inhibited swarm expansion. The strength of effect was contingent upon treatment (treatment effect; *P*=0.012932) and the effect of treatment was contingent upon the time at which the measurement was taken (interaction effect; *P*=0.001932). Swarming occurred consistently earlier for the control than for samples with pesticide treatment. Pyrethrin and malathion had no significant effect. Control (no pesticide) (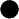); working dilution (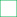); 1 : 10 dilution from working (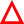); 1 : 100 dilution from working (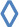).

In order to determine if neem oil could act as a chemoattractant or chemorepellent to alter the swarming motility of *B. subtilis *, we conducted swarm expansion assays using the swarming-proficient *B. subtilis* strain NCIB 3610. We first inoculated concentrated, mid-exponential *B. subtilis* cells onto the swarm agar plate, and then added a drop of neem oil, at three concentrations (working dilution, 1 : 10 and 1 : 100 dilution), midway between the site of inoculation and the edge of the plate. Swarm radii were measured toward and away from the neem oil. There was a ~30 min delay in the initiation of swarm expansion toward the drop of neem oil compared to a control plate without the pesticide (treatment effect, *P*=0.012932) (Fig. 3a). There was also a significant interaction effect (*P*=0.001932). In the generalized linear models used to test for significant differences in swarm expansion, an interaction effect reveals that the lines are of quantitatively different slopes, indicating that at least one of the swarms expanded at a different rate (Figs 3a and S2). Based on the graphical data, the control sample swarmed at a faster rate than the samples with pesticide (Figs 3a and S2). Swarm initiation and movement away from the drop of neem oil were unchanged from control plates without pesticide (data not shown).

The same assay was used to assess the effects of malathion and pyrethrin on swarm expansion. Pyrethrin had no effect on swarm expansion (treatment effect, *P*=0.3574; interaction effect, *P*=0.4114) (Fig. 3b). In the presence of malathion, swarm expansion remained unchanged (treatment effect, *P*=0.11530; interaction effect, *P*=0.06453) (Fig. 3c). We conclude that neither pyrethrin nor malathion act as chemoattractants or chemorepellents in a plate-based assay.

Due to the rapid nature of swarming motility, it is possible that the pesticides might still alter swarming motility, but the cells swarmed too rapidly to respond to the chemicals in the short time course of the assay. To further investigate this possibility, we performed swarm expansion assays in which cells were pretreated with the pesticide for 1 h prior to inoculation onto the swarm agar plate and the pesticides were spread evenly over the surface of the medium to allow maximum contact between the cells and the pesticides. In these assays, swarm expansion was indistinguishable from control plates without pesticide (Fig. S3). In some cases, the swarm appeared to expand more rapidly, but not significantly more so, probably owing to the extra wetness of the agar surface due to spreading the pesticides on top of the agar [12].

### Neem oil alters swarming pattern

In order to further investigate the effect of neem oil on swarming, we applied diluted neem oil as a mist over the surface of a swarm agar plate. Swarms initiated and proceeded similar to controls, but swarm pattern was altered; the normally smooth and regular surfactin ring extending in front of the colony swarm was noticeably interrupted (data not shown). We hypothesized that the increased concentration of neem on the surface of the agar may cause the change in swarm pattern. To test this hypothesis, we spread neem oil on the surface of the agar with glass beads, but the swarm pattern was remained unaltered. Next, we added undiluted neem oil to molten swarm agar prior to the assay; there was a significant effect on swarm expansion rate (treatment effect, *P*=1.127×10^−7^; interaction effect, *P*=9.585×10^−5^). Swarming initiation was delayed when undiluted neem was added to the agar compared to samples containing diluted neem oil (Fig. S4b). While slicks of neem oil could be seen on the top of the agar, neither the surfactin nor the swarm appeared to follow these slicks (data not shown). We conclude that the increased concentration of neem is not responsible for the change in swarm pattern.

We next hypothesized that the oily nature of the neem interacted with the surfactin produced by the swarm, thus altering swarm pattern. To test this hypothesis, we applied mineral oil to the agar. When mineral oil was spread across the surface of swarm agar plates prior to drying, the mineral oil did not spread evenly, and drops of mineral oil could be seen on the top of the agar. *B. subtilis* swarmed rapidly over the surface, but the swarm spread uniformly, and did not spread from oil drop to oil drop (data not shown). When mineral oil was added to molten agar prior to pouring the plates, there was no difference in swarming pattern or rate compared to the wild-type, even when undiluted mineral oil was added (Fig. S4a). We are unable to explain the unusual swarm pattern produced when neem oil is added to swarm agar.

## Discussion

We began this research to investigate whether pesticides acted as chemical signals that would alter the coordinated group behaviours of *B. subtilis *. We found that all three pesticides inhibited biofilm formation. Floating pellicle biofilms in the presence of neem were less textured and thinner than controls, and appeared to develop more slowly. It is possible that the neem oil might either inhibit the bacteria from forming chains and staying in the air–liquid interface – a critical first step in biofilm formation – or slow down such a process [38, 39]. This would account for slowed biofilm formation and the abundance of cell clumps on the bottom of the well at 72 h. Future studies should investigate if exposure to pesticides, particularly neem oil, alters the expression of genes for EPS production.

Inhibition of chain formation, however, cannot account for the difference in colony architecture in the presence of neem oil. In colony architecture assays, the wrinkles and folds found at the centre of the colony are extended outward, compared to colonies without neem oil (Fig. 4a), while floating pellicles exhibit less texturing in the presence of neem compared to controls. It has been shown that, in floating pellicle biofilms, the wrinkling of the biofilm is due to the elastic biofilm growing in a confined space [40]. It may be possible that on a surface, the neem oil disrupts cell spreading, leading to increased folding of the surface biofilm. We note, however, that the size of the colonies with and without neem oil was similar, suggesting that cells spread equally on both plates. Alternatively, wrinkling in surface colony biofilms is thought to be a result of localized cell death [41]. If this is the case, the increase in the proportion of the colony exhibiting wrinkling suggests an increase in dead cells underlying the biofilm. Neem oil did not inhibit growth rate or yield, but some aspect of the biofilm state may make the cells more susceptible to killing by neem oil and cannot be discounted in our studies. More sophisticated analysis will be necessary to determine if the physiology of cells in a biofilm makes them susceptible to killing by neem oil.

While very little difference in biofilm formation was evident in qualitative floating pellicle assays or colony architecture assays with pyrethrin, it did result in an inhibition of biofilm formation at all concentrations tested in quantitative crystal violet assays. It is possible that pyrethrin is interfering with some process unique to biofilm formation at air–liquid interfaces. We hypothesize that decreased wrinkling is the result of either decreased elasticity or altered pellicle thickness [40].

In addition, we are intrigued by the dose-dependent relationship between biofilm formation and malathion concentration. While only the highest concentration of malathion resulted in a change in pellicle appearance, even the most dilute concentration of malathion inhibited biofilm formation as measured by crystal violet assays (Figs 1c and 3c). It is not clear from our studies if it is the malathion or other ingredients in the pesticide formulation that are causing the negative effects. Previous research has noted that petroleum distillates in commercial malathion solutions may impair bacteria more than the malathion itself [42]. It is not clear if it is the other ingredients in the commercial preparation or the malathion that is inhibiting the biofilm. Further investigation into the manner in which malathion-containing pesticides inhibit biofilm formation is needed.

Changes in swarming in response to pesticide were less pronounced. In our plate-based swarming assays, only neem oil acted as a chemorepellent. Even with extended exposure via pretreatment with pesticide, swarming rate was not decreased, indicating that none of the pesticides acted as signals to prevent surfactin production, swarming initiation, or swarming rate.

Neem oil is derived from the fruits and seeds of *Azadirachta indicae*, a tree in the mahogany family [43, 44]. Neem extract contains over 100 active compounds, the best studied of which is azadirachtin, identified in 1968 [43, 45, 46]. *B. subtilis* colonies in the presence of neem oil have a longer lag time before swarming begins than in the absence of neem (Fig. 3a). Because neem is a lipid and the surfactant produced to allow swarming motility is also a lipopeptide, there is a possibility that the surfactin being produced by the colony on the plate will dissolve into the high concentration of neem oil in the media and a much greater amount of surfactin is needed before surface migration can begin. This hypothesis is congruent with observations made during the assay that there was not a perfectly circular surfactin ring preceding the colony as seen in the control group, but rather an irregular, spotty surfactin ring, which could account for the change in swarming pattern (data not shown). However, addition of an oil alone is not sufficient to disrupt swarming pattern, as the same effect was not observed when mineral oil was added. The chemical structures of the oil components of neem and mineral oil differ, and could account for the difference in swarming pattern. How the neem oil is exhibiting its effect at a distance is not clear. Chemotaxis is not required for swarming motility [11], but may alter swarming migration in soils, as *B. subtilis* does chemotax towards plant roots [17], so it is possible that neem is acting as a chemorepellent, though the effect is minor.

Finally, because the growth of *B. subtilis* in the presence of neem oil, malathion, or pyrethrin is not inhibited, we conclude that the pesticides are likely not inhibiting the growth of the bacteria, but rather interrupting or altering some other requirement for biofilm formation and/or swarming motility. We conclude that neem oil has a minor effect on swarming motility, and all three pesticides significantly reduced biofilm formation of *B. subtilis *. We believe that all three pesticides are unlikely to inhibit plant root colonization in garden soils where concentrations of the pesticides are likely to be much lower, but *in situ* studies are needed. Given that some studies have shown a change in soil microbial composition and metabolism in the presence of pesticides [26–32], and the interactions of soil organisms with each other and plants is complex, future investigations into the interactions of pesticides with PGPB are warranted.

## Supplementary Data

Supplementary material 1
