# Supplementary material for: Biofilm formation by Bacillus subtilis is altered in the presence of pesticides
Source: Access Microbiol. 2020 Nov 12;2(12):acmi000175. doi: 10.1099/acmi.0.000175 (PMC7818241; doi:10.1099/acmi.0.000175)
Supplement: Supplementary material 1 [file acmi-2-175-s001.pdf]

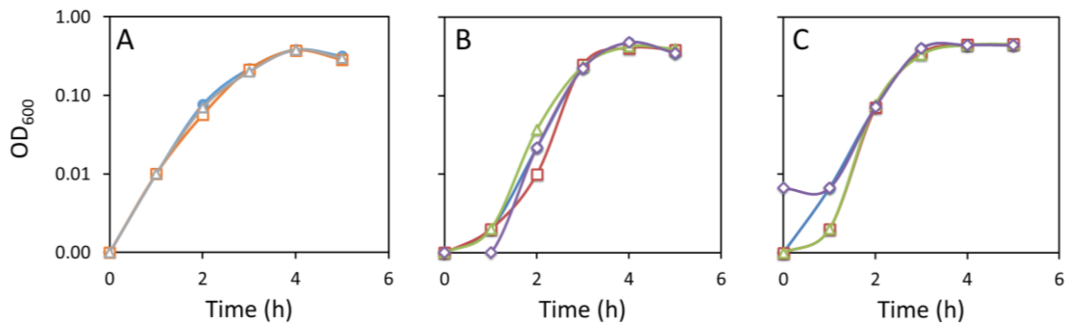

Figure S1. Pesticides do not alter growth rate or yield. Neem oil (A), pyrethrin (B), and malathion (C) were added to a liquid growth media containing *B. subtilis* and the resulting optical density at 600 nm was recorded every hour. Undiluted (○); working dilution (□); 1:10 dilution from working (△); 1:100 dilution from working (◊). All lines represent the average of three replicates.

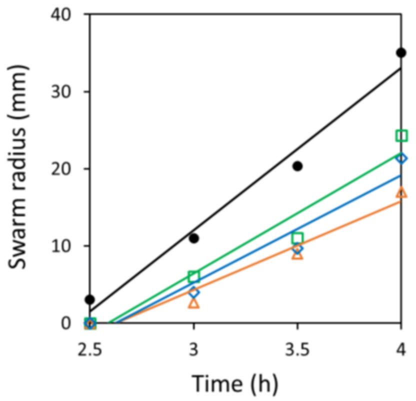

Figure S2. Swarming rate is decreased in the presence of neem oil. Plot of linear portion of swarm expansion assay curves from Figure 3 with lines of best fit to show slope of line during swarm expansion. Slope is equivalent to swarm rate. The control line has a steeper slope (i.e. faster swarm rate) than any of the samples treated with neem oil. Control (no pesticide) (●) (rate = 21.067 mm/hr); working dilution (□) (rate = 15.6 mm.hr); 1:10 dilution from working (△) (rate = 11.467 mm/hr); 1:100 dilution from working (◊) (rate = 13.933 mm/hr).

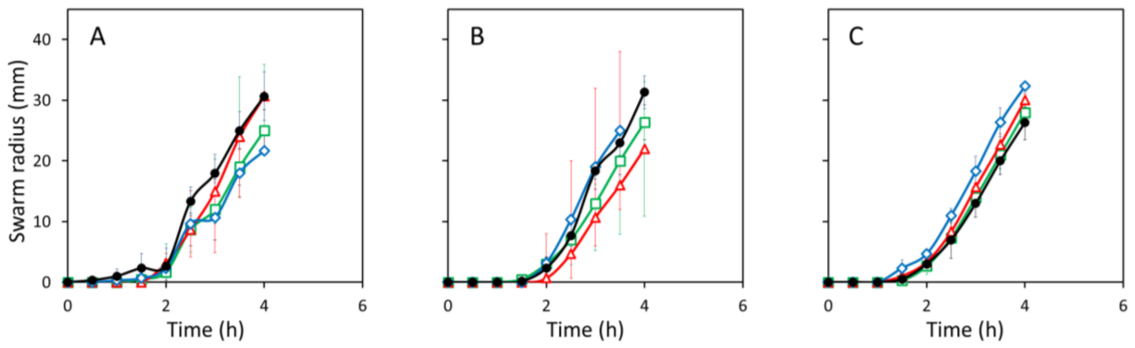

Figure S3. Pretreatment with pesticides does not alter swarm radii. Neem oil (A), pyrethrin (B), and malathion (C) were added to liquid cultures prior to inoculation on swarm plates. 100 µL of pesticides at various concentrations were spread onto a swarming plate and the swarming radii were measured every thirty minutes. All lines represent the average of three replicates. Error bars represent twice the standard error of the mean. No significant effects of treatment or interaction of treatment and time were found. Control (no pesticide) (●); working dilution (□); 1:10 dilution from working (△); 1:100 dilution from working (◊).

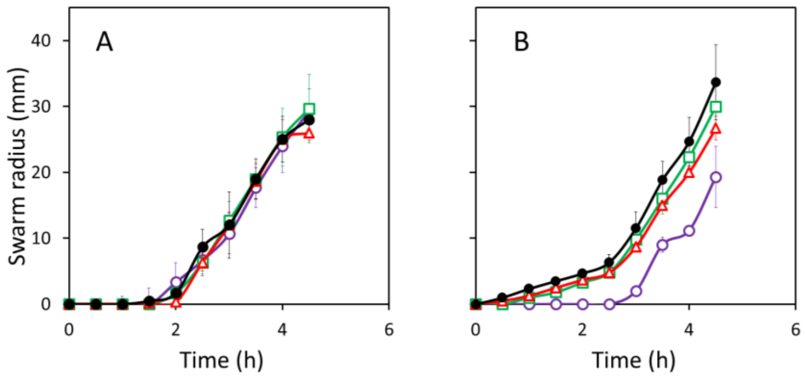

Figure S4. Mineral oil does not alter swarming motility. Mineral oil (A) and neem oil (B) were added to molten swarm agar in various concentrations prior to pouring plates. Swarming colony radii were measured every thirty minutes. All lines represent the average of three replicates. Error bars represent twice the standard error of the mean. No significant effects of treatment or interaction of treatment and time were found with the addition of mineral oil. Undiluted Neem oil caused significant treatment ($p = 0.000000127$) and interaction ($p = 0.0000958$) effects. Control (no additive) (●); undiluted (o); working dilution (□); 1:10 dilution from working (△).
